# Prospective, single-center cohort study analyzing the efficacy of complete laparoscopic resection on recurrent hepatocellular carcinoma

**DOI:** 10.1186/s40880-016-0088-0

**Published:** 2016-03-08

**Authors:** Jie Zhang, Zhong-Guo Zhou, Zhong-Xi Huang, Ke-Li Yang, Jian-Cong Chen, Jin-Bin Chen, Li Xu, Min-Shan Chen, Yao-Jun Zhang

**Affiliations:** Department of Hepatobiliary Oncology, Sun Yat-sen University Cancer Center, State Key Laboratory of Oncology in South China, Collaborative Innovation Center for Cancer Medicine, Guangzhou, 510060 Guangdong P.R. China

**Keywords:** Laparoscopic hepatectomy, Hepatocellular carcinoma, Recurrence, Relapse-free survival

## Abstract

**Background:**

Laparoscopic hepatectomy is increasingly being used to treat hepatocellular carcinoma (HCC). However, few studies have examined the treatment of recurrent HCC in patients who received a prior hepatectomy. The present prospective study compared the clinical efficacy of laparoscopic surgery with conventional open surgery in HCC patients with postoperative tumor recurrence.

**Methods:**

We conducted a prospective study of 64 patients, all of whom had undergone open surgery once before, who were diagnosed with recurrent HCC between June 2014 and November 2014. The laparoscopic group (*n* = 31) underwent laparoscopic hepatectomy, and the control group (*n* = 33) underwent conventional open surgery. Operation time, intraoperative blood loss, surgical margins, postoperative pain scores, postoperative time until the patient could walk, anal exsufflation time, length of hospital stay, and inpatient costs were compared between the two groups. The patients were followed up for 1 year after surgery, and relapse-free survival was compared between the two groups.

**Results:**

All surgeries were successfully completed. No conversion to open surgery occurred in the laparoscopic group, and no serious postoperative complications occurred in either group. No significant difference in inpatient costs was found between the laparoscopic group and the control group (*P* = 0.079), but significant differences between the two groups were observed for operation time (116.7 ± 37.5 vs. 148.2 ± 46.7 min, *P* = 0.031), intraoperative blood loss (117.5 ± 35.5 vs. 265.9 ± 70.3 mL, *P* = 0.012), postoperative time until the patient could walk (1.6 ± 0.6 vs. 2.2 ± 0.8 days, *P* < 0.05), anal exsufflation time (2.1 ± 0.3 vs. 2.8 ± 0.7 days, *P* = 0.041), visual analogue scale pain score (*P* < 0.05), postoperative hepatic function (*P* < 0.05), and length of hospital stay (4.5 ± 1.3 vs. 6.0 ± 1.2 days, *P* = 0.014). During the 1-year postoperative follow-up period, 6 patients in each group had recurrent HCC on the side of the initial operation, but no significant difference between groups was observed in the recurrence rate or relapse-free survival. In the laparoscopic group, operation time, postoperative time until the patient could walk, anal exsufflation time, and inpatient costs were not different (*P* > 0.05) between the patients with contralateral HCC recurrence (*n* = 18) and those with ipsilateral HCC recurrence (*n* = 13). However, intraoperative blood loss was significantly less (97.7 ± 14.0 vs. 186.3 ± 125.6 mL, *P* = 0.012) and the hospital stay was significantly shorter (4.2 ± 0.7 vs. 6.1 ± 1.7 days, *P* = 0.021) for the patients with contralateral recurrence than for those with ipsilateral recurrence.

**Conclusions:**

For the patients who previously underwent conventional open surgical resection of HCC, complete laparoscopic resection was safe and effective for recurrent HCC and resulted in a shorter operation time, less intraoperative blood loss, and a faster postoperative recovery than conventional open surgery. Laparoscopic resection was especially advantageous for the patients with contralateral HCC recurrence.

## Background

Liver cancer is a common cancer in China, particularly in men and residents of rural areas [1, 2]. Postoperative recurrent hepatocellular carcinoma (HCC) presents a challenge for surgeons. Currently, radiofrequency ablation is an effective treatment for HCCs that are smaller than 3 cm [3–6]; transcatheter arterial chemoembolization (TACE) combined with computed tomography-guided percutaneous thermal ablation can also be used to treat these tumors [7]. However, in most cases, radiofrequency ablation is contraindicated in patients with recurrent HCC because of large tumor size, severe peritoneal adhesions, or tumor location (at the surface of the liver and adjacent to large vessels). TACE is generally a non-radical treatment; repeat surgical resection is an effective radical treatment. In the past, open surgery was often performed, which had disadvantages such as a large incision, a complex surgical approach, and a slow recovery. The development of laparoscopic techniques has produced advances in the complete laparoscopic resection of recurrent HCC [7–9], and single-entry laparoscopic resection and radiofrequency ablation combined with laparoscopic resection of recurrent HCC have been reported [10]. However, further research is needed to evaluate the treatment outcomes of these operations. We conducted a prospective study of 64 patients who underwent resection of recurrent HCC at our hospital between June 2014 and November 2014 and performed a comparative evaluation of laparoscopic resection and open surgery.

## Methods

### Patients and inclusion/exclusion criteria

The Institutional Review Board at Sun Yat-sen University Cancer Center approved this prospective study, and the methods were carried out in accordance with approved guidelines (ClinicalTrials.gov ID: NCT02613156).

The inclusion criteria were as follows: (1) recurrent HCC after open surgery; (2) recurrent HCC located in any part of the left lateral lobe or the diaphragm side of the right lobe and near the surface of the liver, without noteworthy surgical contraindications; (3) no major vessel or bile duct tumor invasion or metastasis; (4) grade A or B liver function or grade C liver function that recovered to grade A after liver-protective treatment; and (5) a signed informed consent form from the patient. The exclusion criteria were as follows: (1) major vessel or bile duct tumor invasion; (2) recurrent HCC located in the right liver parenchyma and near secondary vessels and bile ducts; (3) extrahepatic metastasis; (4) grade C liver function; (5) noteworthy surgical contraindications; or (6) patient refusal to undergo laparoscopic hepatectomy.

### Observation indicators and detection methods

Routine biochemical tests were conducted as part of the postoperative re-examination, and changes in alanine aminotransferase and aspartate aminotransferase levels were used to assess postoperative liver function changes. The severity of adhesions was assessed using the modified American Fertility Society (mAFS) classification scoring system. Pain scores were evaluated using a visual analogue scale (VAS) after surgery (the 1st day). Patients were followed up every 2 or 3 months after discharge from the hospital.

### Treatments

#### Surgical instruments

The surgical instruments used for patients in the laparoscopic group included conventional laparoscopic instruments (three 5-mm trocars, two 10-mm trocars, and two damage-free laparoscopic clamps), the STORZ high-resolution laparoscopic operating system (Mittelstra*ße*, Tuttlingen, Germany), the GEN300 ultrasonic scalpel system (Johnson and Johnson, New Brunswick, NJ, USA), a laparoscopic linear cutter stapler (Johnson and Johnson, New Brunswick, NJ, USA), a laparoscopic ultrasound device, LigaSure, bipolar coagulation, titanium laparoscopic clips, and absorbable hemostatic gauze.

The surgical instruments used for patients in the control group included conventional laparotomy instruments, an electric scalpel, an intraoperative B-mode ultrasound device, proline tubing, gelatin sponges, and absorbable hemostatic gauze.

#### Surgical approaches

All patients received general anesthesia and intubation. Based on the tumor location, patients in the laparoscopic group were placed in the supine position, with their legs spread apart or horizontally; patients in the control group were positioned horizontally. Moreover, in the laparoscopic group, if the tumor was in the right lobe, a five-way-access approach was used, in which the surgeon stood on the same side as the tumor, the laparoscope-supporting assistant stood between the patient’s legs, and the surgical assistant stood on the other side of the tumor. A small incision (1.5 cm in diameter) was made along the upper navel edge, and a Veress needle was inserted to establish pneumoperitoneum. Next, a 10-mm trocar was inserted, and then the laparoscope was inserted to explore the liver tumor and peritoneal adhesions. Pressure was maintained at 8–12 mmHg. Next, a small incision (0.5 cm in diameter) was made 2 cm underneath the right costal margin, and a 5-mm trocar was inserted for auxiliary surgical access. Another small incision (1.0 cm in diameter) was made laterally and inferiorly, and a 10-mm trocar was inserted and used as the main surgical access. A small incision (0.5 cm in diameter) was then made underneath the left costal margin, and a 5-mm trocar was inserted for auxiliary surgical access. These access points were located as far away from the adhesions as possible. If it was impossible to circumvent peritoneal adhesions, laparoscopic separation of the adhesions was performed first. If the tumor was in the S2 or S3 segment, a four-way-access approach was used: the surgeon stood on the right side of the patient, and the first assistant stood on the left side of the patient. Only one access point underneath the left costal margin was required. During resection of tumor lesions and liver segments from the right lobe, the surgeon separated adhesions in the operating field that were near the abdominal wall using an ultrasonic scalpel and then determined the location and incision edges of the tumor under the guidance of laparoscopic ultrasound. Next, the surgeon used an ultrasound-guided scalpel to separate the liver parenchyma, followed by the use of LigaSure coagulation to stop bleeding from small vessels. Groups of titanium clips were used to stop the bleeding if the LigaSure coagulation was ineffective. The surgeon resected the tumor after separating the liver parenchyma. If the tumor was in the S2 or S3 segment, the surgeon performed laparoscopic resection of the left lateral lobe. After separating the adhesions, the surgeon cut the left coronary ligament and the left triangular ligament. The assistant pulled the left lobe upward and to the right, and the surgeon separated the hepatogastric ligament and verified that the incision edge was more than 2 cm away from the tumor (guided by laparoscopic ultrasound). Next, the surgeon separated the liver parenchyma along the incisions on the surface of the liver until reaching the vascular pedicle of the S2 and S3 segments and then used a laparoscopic linear cutter stapler to cut the residual liver tissue and vessels to resect the left lobe. A gelatin sponge was used to stop minor bleeding in the operating field. After the wound was checked for active bleeding, the surgeon expanded the incision at the navel edges to remove the specimen. Next, the surgeon placed an abdominal drainage tube according to standard procedures. For patients in the control group, if the tumor was in the left lateral lobe or the right anterior lobe, an incision was made along the midline of the abdominal wall; if the tumor was in the right lobe, an oblique incision was made underneath and along the costal margin. Adhesions were separated after the abdominal wall was opened, and intraoperative B-mode ultrasound was used to determine the location of the tumor and incision lines. A conventional surgical approach was used for local resection or segmental resection of the HCC. If the tumor was in the S2 or S3 segment, the left lateral lobe was resected with an open lobectomy, and an abdominal drainage tube was placed according to standard procedures.

### Statistical analysis

SPSS 21.0 software was used to process all data. Measurement data are expressed as the mean ± standard deviation and were analyzed with an independent-sample *t* test. Count data were analyzed with the *χ*^2^ test. Relapse-free survival was analyzed using the Kaplan–Meier method. *P* < 0.05 were considered statistically significant.

## Results

### Study population and baseline clinical characteristics

We studied 64 patients who underwent resection of recurrent HCC at our hospital between June 2014 and November 2014. The laparoscopic group comprised 31 patients who underwent laparoscopic resection of the recurrent HCC; the control group comprised 33 patients who underwent conventional open surgery. Case grouping was decided by a multidisciplinary team. In the laparoscopic group, 26 patients were men and 5 patients were women (age range, 37–66 years; median, 54 years); the recurrent tumors had an average size of 2.5 ± 1.0 cm. In the control group, 27 patients were men and 6 patients were women (age range, 34–65 years; median, 59.5 years); the recurrent tumors had an average size of 3.8 ± 1.1 cm. The hepatic falciform ligament comprised the border between the left lateral lobe (to the left) and the right lobe (to the right). Detailed clinical data of the two groups of patients are shown in Table 1. No significant difference was observed between the two groups of patients in their general information before the operation.

**Table 1:** Baseline characteristics of all enrolled patients

Characteristic	Laparoscopic group	Open surgery group (control)	*P* value
Sex (cases)			0.904
Men	26	27	
Women	5	6	
Age (years)			0.413
Median	54	59.5	
Range	37–66	34–65	
BMI			0.912
Median	24.4	25.0	
Range	19.1–31.0	21.8–29.9	
Initial surgical lesion site (cases)			0.665
Right lobe	22	25	
Left lobe	9	8	
Primary tumor size (cm)	4.1 ± 1.5	4.9 ± 2.6	0.382
Time to recurrence (months)	28.4 ± 27.7	27.5 ± 16.3	0.282
Size of recurrent tumor (cm)	2.5 ± 1.0	3.8 ± 1.1	0.45
Recurrent site (cases)			0.632
Right lobe	16	19	
Left lobe	15	14	
Operation method (cases)			0.955
Left lateral lobectomy	12	13	
Local resection	19	20	
Total bilirubin (μmol/L)	31.2 ± 7.4	33.7 ± 10.4	0.742
Albumin (g/L)	35.2 ± 3.3	36.4 ± 4.3	0.532
ALT (U/L)	49.6 ± 6.4	76.3 ± 7.5	0.671
AST (U/L)	52.4 ± 7.4	69.4 ± 8.4	0.832
Prothrombin time (s)	11.5 ± 0.7	12.4 ± 1.0	0.651
AFP (cases)			0.264
>400 ng/mL	27	24	
<400 ng/mL	4	9	
HBV-DNA (log10 IU/mL)	3.50 ± 0.8	3.0 ± 0.2	0.192

*BMI* body mass index, *ALT* alanine aminotransferase, *AST* aspartate aminotransferase, *AFP* α-fetoprotein, *HBV* hepatitis B virus

There were 53 men and 11 women patients, with a median age of 52.5 years (range, 45–74 years). Except for hepatectomy, 9, 5, and 2 patients underwent further TACE, local thermal ablation, and sorafenib treatment, respectively, before this study. The mean baseline characteristics were similar between the two groups. The median follow-up period for all patients was 17 months (range, 12–18 months); the median survival time was 11.0 months (95% confidence interval 9.2–15.9 months).

### Efficacy of complete laparoscopic resection

All operations were successfully completed, and none of the patients in the laparoscopic group required conversion to open surgery. Moreover, no serious postoperative complications were observed in either group. Significant differences were observed between the laparoscopic and control groups in operation time (116.7 ± 37.5 vs. 148.2 ± 46.7 min, *P* = 0.031), intraoperative blood loss (117.5 ± 35.5 vs. 265.9 ± 70.3 mL, *P* = 0.012), postoperative time until the patient could walk (1.6 ± 0.6 vs. 2.2 ± 0.8 days, *P* = 0.004), anal exsufflation time (2.1 ± 0.3 vs. 2.8 ± 0.7 days, *P* = 0.041), VAS scores (*P* < 0.001), postoperative hepatic function (*P* < 0.05), and length of hospital stay (4.5 ± 1.3 vs. 6.0 ± 1.2 days, *P* = 0.014). In contrast, no differences between the two groups were observed in surgical margins, grade of peritoneal adhesions, or inpatient costs (Table 2).

**Table 2:** Comparison of perioperative conditions between the laparoscopic and control groups

Characteristic	Laparoscopic group	Open surgery group	*P* value
Operation time (min)	116.7 ± 37.5	148.2 ± 46.7	0.031
Surgical margin (cm)	2.1 ± 1.2	2.2 ± 0.6	0.068
Intraoperative blood loss (mL)	117.5 ± 35.5	265.9 ± 70.3	0.012
Grade of peritoneal adhesions (cases)			0.880
1	18	20	
2	8	6	
3	3	4	
4	2	3	
VAS score (cases)			<0.001
0–3	20	9	
4–6	8	18	
7–10	3	6	
ALT (U/L)^a^	179.6 ± 17.4	312.3 ± 28.2	0.012
AST (U/L)^a^	84.1 ± 16.1	223.4 ± 30.1	0.029
Anal exsufflation time (days)	2.1 ± 0.3	2.8 ± 0.7	0.041
Postoperative time until the patient could walk (days)	1.6 ± 0.6	2.2 ± 0.8	0.004
Hospital stay (days)	4.5 ± 1.3	6.0 ± 1.2	0.014
Inpatient costs (×10,000 yuan)	6.3 ± 0.4	5.5 ± 0.6	0.079
Postoperative recurrence [cases (%)]	5 (16.1)	7 (21.2)	0.603

*VAS* visual analogue scale, *BMI* body mass index, *ALT* alanine aminotransferase, *AST* aspartate aminotransferase, *AFP* α-fetoprotein, *HBV* hepatitis B virus

^a^ALT and AST were tested on the 3rd day after surgery

### Comparison of patients with contralateral and ipsilateral recurrence after laparoscopic hepatectomy

Moreover, we compared the data for patients in the laparoscopic group with contralateral or ipsilateral recurrence (18 and 13 patients, respectively, based on the initial operation). Interestingly, compared with patients with ipsilateral recurrence, patients with contralateral recurrence tended to have less intraoperative blood loss (97.7 ± 14.0 vs. 186.3 ± 125.6 mL, *P* = 0.012) and a shorter hospital stay (4.2 ± 0.7 vs. 6.1 ± 1.7 days, *P* = 0.021) (Table 3).

**Table 3:** Comparison of patients with contralateral and ipsilateral recurrence after laparoscopic hepatectomy

Characteristic	Contralateral recurrence (*n* = 18)	Ipsilateral recurrence (*n* = 13)	*P* value	*T*/*χ* ^2^
Recurrent tumor size (cm)	4.4 ± 0.9	3.7 ± 1.4	0.227	1.3
Operation time (min)	112.4 ± 18.6	159.4 ± 39.0	0.034	−0.6
Surgical margin (cm)	3.6 ± 1.0	2.1 ± 0.6	0.166	−0.3
Intraoperative blood loss (mL)	97.7 ± 14.0	186.3 ± 125.6	0.012	−2.1
Postoperative time until the patient could walk (days)	1.8 ± 0.5	1.6 ± 0.5	0.478	−0.5
Anal exsufflation time (days)	2.3 ± 0.5	2.1 ± 0.7	0.634	−0.2
Hospital stay (days)	4.2 ± 0.7	6.1 ± 1.7	0.021	−2.1
Inpatient costs (×10,000 yuan)	6.2 ± 0.3	5.8 ± 0.7	0.575	−0.6

### Short-term effect of laparoscopic hepatectomy on recurrent HCC

**Fig. 1:**
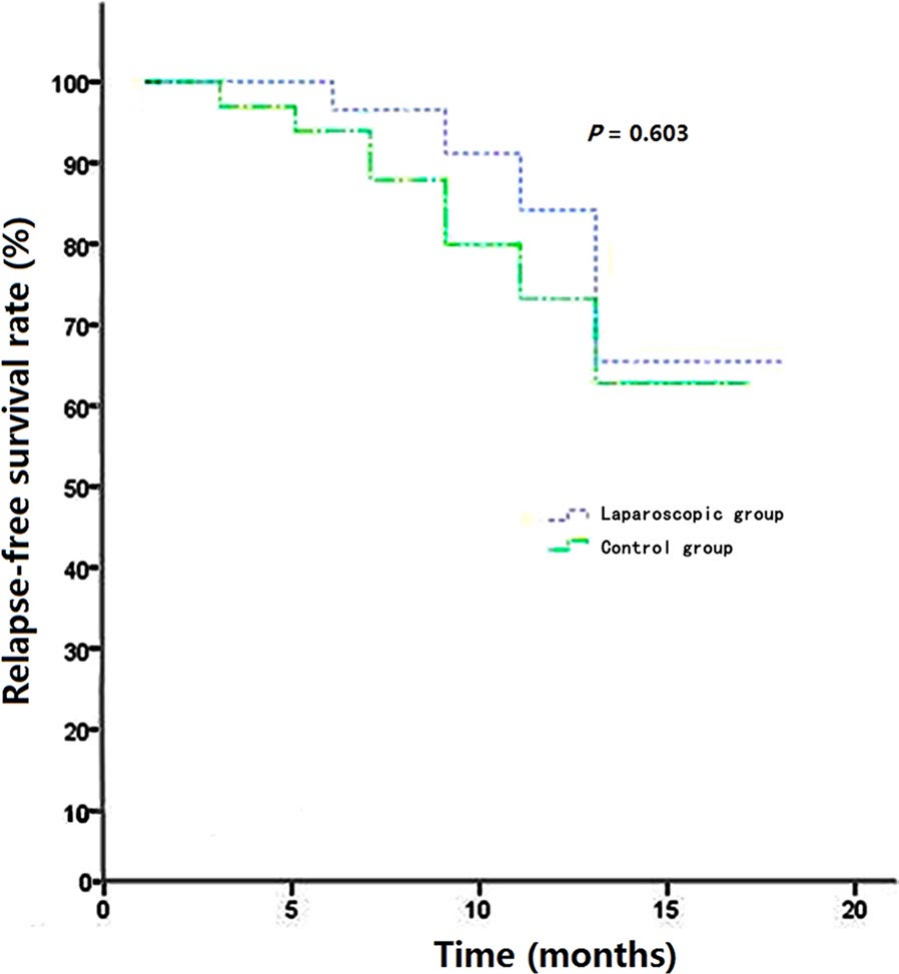
Kaplan–Meier *curves* showing relapse-free survival (RFS) for all 64 patients with hepatocellular carcinoma. No significant difference in RFS was observed between the laparoscopic group and the open surgery (control) group

During the 1-year postoperative follow-up period, 5 patients (16.1%) in the laparoscopic group and 7 patients (21.2%) in the control group relapsed, although no significant difference was observed in relapse-free survival between the two groups (Fig. 1).

## Discussion

Based on the results of this study, we propose that laparoscopic resection of recurrent HCC is feasible for patients who previously underwent conventional open surgery. Compared with conventional open surgery, complete laparoscopic resection of recurrent HCC has the advantages of a shorter operation time, less intraoperative blood loss, and less pain, resulting in a faster postoperative recovery and a shorter hospital stay.

Since Reich et al. [11] reported the first successful laparoscopic liver resection in 1991, the use of laparoscopy for liver resection has gradually increased. Additionally, laparoscopic liver surgery has become increasingly common as laparoscopic surgical instruments have improved and surgeons have gained more experience [12–16]. Several studies have reported laparoscopic resection of the caudate lobe and complete laparoscopic resection combined with associated liver partition and portal vein ligation for staged hepatectomy (ALPPS) [17–20]. Moreover, pilot studies have confirmed the feasibility and value of complete laparoscopic ALPPS, and ALPPS has been used to remove donor livers during liver transplantation [21–24].

Generally, because of the complexity of postoperative adhesion, laparoscopic surgery is not recommended for repeat surgery, and there have been only a few reports on this approach. Kanazawa et al. [8] and Chan et al. [9] reported their results for laparoscopic resection of recurrent HCC; they found that patients in the laparoscopic group had significantly less intraoperative blood loss than patients in the open surgery group, which was consistent with the results of our study. In addition, we found that, regarding operation time, postoperative time until the patient could walk, postoperative pain, and length of hospital stay, laparoscopic resection was superior to open surgery. Patients in the laparoscopic group had a shorter operation time for the following reasons: (1) once pneumoperitoneum was established under laparoscopic guidance, the increase in pressure increased the tension of adhesions, which, together with a large laparoscopic operating field, facilitated the separation of the adhesions; (2) certain adhesions in non-operating fields could be circumvented by laparoscopic instruments and thus required no separation, resulting in no effect on exposure or the operation; and (3) during resection of the left lateral lobe, a cutting and closure device was typically used, eliminating the need to separate segmental vessels, thereby greatly reducing the operation time and bleeding. Moreover, the pressure of the pneumoperitoneum itself played a role in hemostasis. Intestinal adhesions were observed in both the laparoscopic group and the control group. The adhesions were rated as 4 or lower (according to the mAFS scoring system), and no extremely severe adhesions (5 or higher) were observed in this study, which highlighted the advantages of using a laparoscope, aided by large operating fields and the pressure produced by pneumoperitoneum, to separate mild to moderate adhesions. Patients who underwent laparoscopic resection of recurrent HCC had significantly lower postoperative pain scores, mainly because of markedly less nerve damage to the abdominal wall due to the use of smaller access points than those used for conventional large incisions. Moreover, because laparoscopic resection caused fewer injuries than open surgery, the patients could walk sooner, and their bowel functions recovered faster after the operation, thereby shortening the hospital stay. Nevertheless, no significant difference in inpatient costs was observed between the two groups.

During the 1-year follow-up period, no patients in either group died. Six patients in each group had recurrent HCC on the same side as the initial operation and underwent interventional treatment or radiofrequency ablation. No significant difference was observed in relapse-free survival between the two groups. A subgroup analysis of patients who underwent laparoscopic resection showed that intraoperative blood loss was significantly less and the hospital stay was significantly shorter for patients who had contralateral recurrence than for patients who had ipsilateral recurrence. For patients who had contralateral recurrence, milder adhesions in the operating field helped to reduce bleeding during the separation of peritoneal adhesions, thereby shortening the operation time, accelerating postoperative recovery, and highlighting the advantages of laparoscopic resection.

## Conclusions

We found that laparoscopic resection of HCC is more effective than open surgery, and we recommend that it may be more widely used in clinical practice, even in cases of recurrent HCC. Complete laparoscopic resection is especially advantageous for recurrent lesions near the surface of the liver, such as the edges of the contralateral lobe, left lobe, and right lobe, and may be used as the preferred treatment. However, this was a non-randomized study, and a randomized controlled study with a large sample size is needed to confirm our results.

## References

[1] Zuo TT, Zheng RS, Zhang SW, Zeng HM, Chen WQ (2015). Incidence and mortality of liver cancer in China in 2011. Chin J Cancer.

[2] Wei KR, Yu X, Zheng RS, Peng XB, Zhang SW, Ji MF (2014). Incidence and mortality of liver cancer in China, 2010. Chin J Cancer.

[3] Peng ZW, Liu FR, Ye S, Xu L, Zhang YJ, Liang HH (2013). Radiofrequency ablation versus open hepatic resection for elderly patients (>65 years) with very early or early hepatocellular carcinoma. Cancer.

[4] Peng ZW, Zhang YJ, Chen MS, Xu L, Liang HH, Lin XJ (2013). Radiofrequency ablation with or without transcatheter arterial chemoembolization in the treatment of hepatocellular carcinoma: a prospective randomized trial. J Clin Oncol.

[5] Gao HJ, Chen MS (2012). Radiofrequency ablation therapy and its strategies in multidisciplinary treatment of hepatocellular carcinoma. Zhonghua Gan Zang Bing Za Zhi.

[6] Chen MS, Peng ZW, Xu L, Zhang YJ, Liang HH, Li JQ (2011). Role of radiofrequency ablation in the treatment of hepatocellular carcinoma: experience of a cancer center in China. Oncology.

[7] Li S, Zhang L, Huang ZM, Wu PH (2015). Transcatheter arterial chemoembolization combined with CT-guided percutaneous thermal ablation versus hepatectomy in the treatment of hepatocellular carcinoma. Chin J Cancer.

[8] Kanazawa A, Tsukamoto T, Shimizu S, Kodai S, Yamamoto S, Yamazoe S (2013). Laparoscopic liver resection for treating recurrent hepatocellular carcinoma. J Hepatobiliary Pancreat Sci.

[9] Chan AC, Poon RT, Chok KS, Cheung TT, Chan SC, Lo CM (2014). Feasibility of laparoscopic re-resection for patients with recurrent hepatocellular carcinoma. World J Surg.

[10] Tsuchiya M, Otsuka Y, Maeda T, Ishii J, Tamura A, Kaneko H (2012). Efficacy of laparoscopic surgery for recurrent hepatocellular carcinoma. Hepatogastroenterology.

[11] Reich H, McGlynn F, DeCaprio J, Budin R (1991). Laparoscopic excision of benign liver lesions. Obstet Gynecol.

[12] Cannon RM, Brock GN, Marvin MR, Buell JF (2011). Laparoscopic liver resection: an examination of our first 300 patients. J Am Coll Surg.

[13] Kingham TP, D’Angelica MI, Jarnagin WR (2012). Laparoscopic liver resection. J Am Coll Surg.

[14] Nomi T, Fuks D, Agrawal A, Kawaguchi Y, Ogiso S, Gayet B (2015). Totally laparoscopic right hepatectomy combined with resection of the inferior vena cava by anterior approach. Ann Surg Oncol.

[15] Cai X, Li Z, Zhang Y, Yu H, Liang X, Jin R (2014). Laparoscopic liver resection and the learning curve: a 14-year, single-center experience. Surg Endosc.

[16] Dulucq JL, Wintringer P, Stabilini C, Mahajna A (2006). Isolated laparoscopic resection of the hepatic caudate lobe: surgical technique and a report of 2 cases. Surg Laparosc Endosc Percutan Tech.

[17] Nakahira S, Takeda Y, Katsura Y, Kato T, Hatanaka N, Tamura S (2014). Laparoscopic left hepatectomy with tumor thrombectomy in patients with hepatocellular carcinoma concomitant with advanced portal vein tumor thrombus. Surg Endosc.

[18] Kyriakides C, Panagiotopoulos N, Jiao LR (2012). Isolated laparoscopic caudate lobe resection. Surg Laparosc Endosc Percutan Tech.

[19] Xiao L, Li JW, Zheng SG (2015). Totally laparoscopic ALPPS in the treatment of cirrhotic hepatocellular carcinoma. Surg Endosc.

[20] Cai X, Peng S, Duan L, Wang Y, Yu H, Li Z (2014). Completely laparoscopic ALPPS using round-the-liver ligation to replace parenchymal transection for a patient with multiple right liver cancers complicated with liver cirrhosis. J Laparoendosc Adv Surg Tech A.

[21] Machado MA, Makdissi FF, Surjan RC (2012). Totally laparoscopic ALPPS is feasible and may be worthwhile. Ann Surg.

[22] Samstein B, Cherqui D, Rotellar F, Griesemer A, Halazun KJ, Kato T (2013). Totally laparoscopic full left hepatectomy for living donor liver transplantation in adolescents and adults. Am J Transplant.

[23] Troisi RI, Elsheikh YM, Shagrani MA, Broering D (2014). First fully laparoscopic donor hepatectomy for pediatric liver transplantation using the indocyanine green near-infrared fluorescence imaging in the Middle East: a case report. Ann Saudi Med.

[24] Rotellar F, Pardo F, Benito A, Marti-Cruchaga P, Zozaya G, Lopez L (2013). Totally laparoscopic right-lobe hepatectomy for adult living donor liver transplantation: useful strategies to enhance safety. Am J Transplant.

